# Reducing annotation effort in agricultural data: simple and fast unsupervised coreset selection with DINOv2 and K-means

**DOI:** 10.3389/fpls.2025.1546756

**Published:** 2025-05-14

**Authors:** Laura Gómez-Zamanillo, Nagore Portilla, Artzai Picón, Itziar Egusquiza, Ramón Navarra-Mestre, Andoni Elola, Arantza Bereciartua-Perez

**Affiliations:** ^1^ TECNALIA, Basque Research and Technology Alliance, Parque Tecnológico de Bizkaia, Derio, Bizkaia, Spain; ^2^ University of the Basque Country, Bilbao, Bizkaia, Spain; ^3^ BASF SE, Limburgerhof, Germany

**Keywords:** label-efficient learning, coreset selection, foundation models, agriculture, unsupervised clustering

## Abstract

The need for large amounts of annotated data is a major obstacle to adopting deep learning in agricultural applications, where annotation is typically time-consuming and requires expert knowledge. To address this issue, methods have been developed to select data for manual annotation that represents the existing variability in the dataset, thereby avoiding redundant information. Coreset selection methods aim to choose a small subset of data samples that best represents the entire dataset. These methods can therefore be used to select a reduced set of samples for annotation, optimizing the training of a deep learning model for the best possible performance. In this work, we propose a simple yet effective coreset selection method that combines the recent foundation model DINOv2 as a powerful feature selector with the well-known K-Means clustering method. Samples are selected from each calculated cluster to form the final coreset. The proposed method is validated by comparing the performance metrics of a multiclass classification model trained on datasets reduced randomly and using the proposed method. This validation is conducted on two different datasets, and in both cases, the proposed method achieves better results, with improvements of up to 0.15 in the F1 score for significant reductions in the training datasets. Additionally, the importance of using DINOv2 as a feature extractor to achieve these good results is studied.

## 1 Introduction

Over the past decade, deep learning has significantly boosted computer vision, enabling the development of solutions for scenarios where traditional image processing methods fell short. However, a notable drawback of deep learning lies in its demand for substantial amounts of annotated data to train models effectively. This poses a challenge for adopting these techniques in fields like agriculture, where annotating data involves considerable effort and specialized expertise.

In many agricultural scenarios, data availability isn’t the issue—data acquisition is straightforward, and there are many examples to collect. The expensive part lies in manually annotating that data. To address this, researchers are recently exploring methods to reduce the required amount of annotated data (Li et al., 2023; Hu et al., 2021; Zhu et al., 2020; Shorewala et al., 2021; Egusquiza et al., 2022; Argüeso et al., 2020). Some of these methods involve selecting specific samples for annotation from all the available data. The underlying idea is that training a deep learning model is more efficient when we have a smaller set of high-quality annotated data that accurately represents the existing variability, rather than a large, annotated dataset with many similar samples that may not provide additional information to the model (Roscher et al., 2023; Wang et al., 2022). However, selecting representative samples in a completely unsupervised and straightforward manner remains a challenge in agriculture, where data content is highly specific to the field. Effectively extracting relevant information from images in an unsupervised and simple way, without resorting to complex methods, is difficult in agricultural data but would be highly beneficial.

In this work, we address the critical task of selecting a set of representative samples for annotation to use in the development of a new agricultural deep learning model. We propose a straightforward and totally unsupervised method to identify the most valuable samples that are worth annotating from a large pool of unlabeled data. Our focus is on creating a small, representative subset of data that captures the essential information present in the entire available dataset—a process commonly referred to as coreset selection. Given that annotation resources are often limited in practical scenarios, our goal is to achieve optimal results within these constraints.

Coreset selection has already been used in the context of selecting training data for machine learning models. Bachem et al. (2017) provided an overview of coreset selection techniques and analyzed the results for several common machine learning problems. Borsos et al. (2020) presented a coreset selection method based on cardinality-constrained bilevel optimization in the context of maintaining data summaries to avoid the catastrophic forgetting in continual learning scenarios. In (Killamsetty et al., 2021) the authors presented RETRIEVE, a coreset selection method for making semi-supervised learning trainings more efficient by selecting a set of unlabeled data to use. CRAIG (Mirzasoleiman et al., 2020) is a coreset selection method based on gradient matching. Lee et al. (2024) proposes a coreset selection method oriented to object detection based on the generation of image-wise and class-wise representative feature vectors for multiple objects of the same class within each image. In (Guo et al., 2022) the authors presented DeepCore, a comprehensive library specifically designed to facilitate the implementation of coreset selection techniques in deep neural networks. In addition, they provided a survey and comparison of the most popular methods in the CIFAR10 and ImageNet databases. One interesting conclusion of this work is that they verify that, considering the methods compared, random selection reduction remained a great baseline.

In our coreset selection approach, we first extract relevant features from the dataset images and then apply an unsupervised clustering algorithm like K-means, clustering similar samples together and separating diverse ones. By selecting samples from each cluster, we create a coreset that effectively represents the dataset’s essential variability. Our proposed method is simple and easy to implement in any agricultural use case, relying on effectively extracting relevant features from the available data in an unsupervised manner. For this task, we evaluate the use of a state-of-the-art large foundation model, which excels at extracting high-quality features from images of a specific application field like agriculture.

We demonstrate the effectiveness of our method to select representative samples in two public agricultural datasets with unbalanced classes. Additionally, we highlight the relevance of DINOv2 (Oquab et al., 2023) in our method, the best image foundation model to date, which significantly improves upon previous pre-trained model backbones in the extraction of relevant features.

The paper is organized as follows. After an introduction to the problem, the datasets are explained in Section 2. A detailed description of the proposed solution is detailed in Section 3. Section 4 explains the experiments performed to validate the method. The results are gathered and discussed in Section 5, and conclusions are summarized in section 6.

## 2 Materials

In this study, we use two public agricultural datasets. The purpose of using these two datasets is to validate the proposed method across two different agricultural use cases, each with varying numbers of images and classes, as well as different types of content within the images. We choose one dataset with a lot of images and few classes and another dataset with less images and a big number of classes.

### 2.1 *PlantVillage* dataset

**Figure 1:**
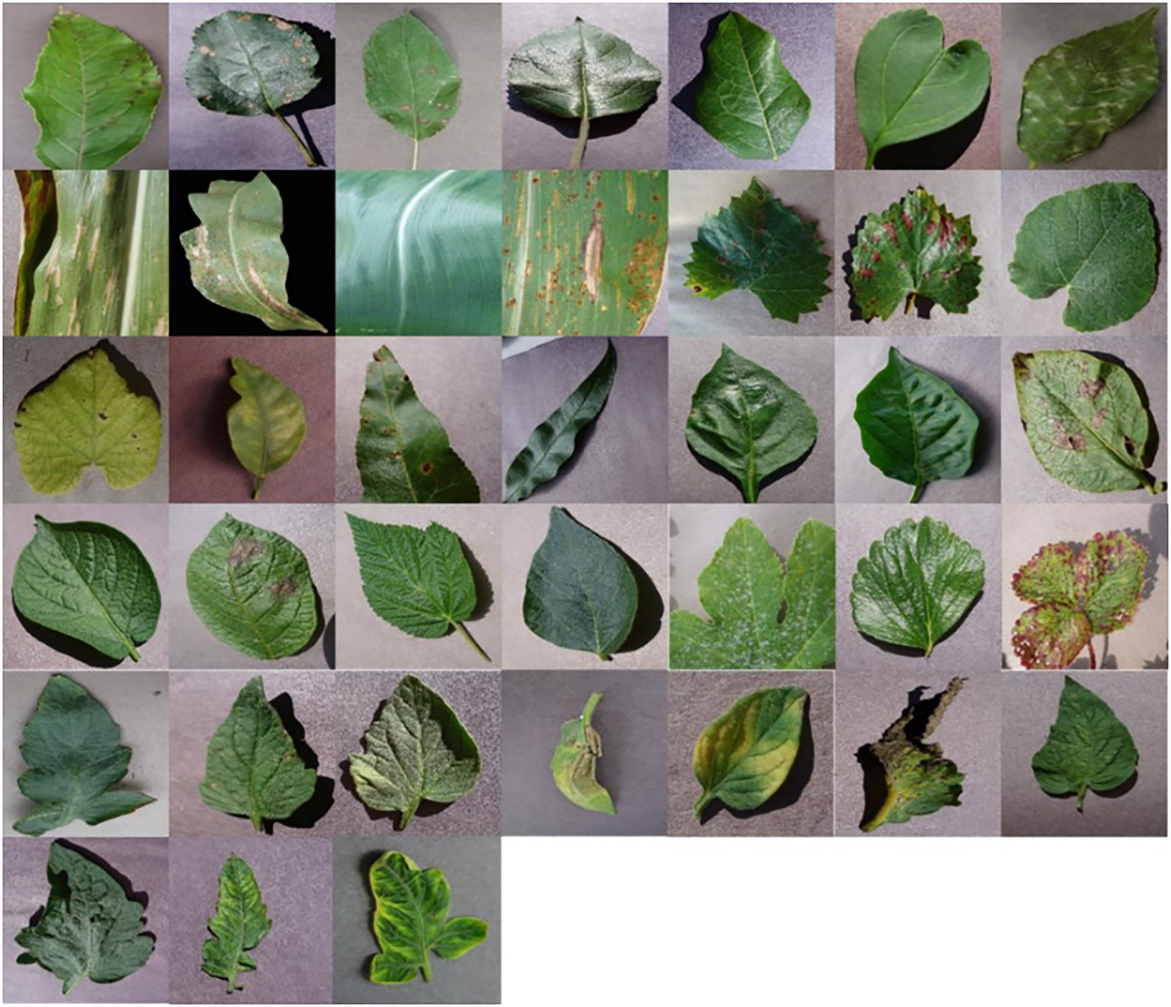
Examples from the 38 classes in the *PlantVillage* (Hughes and Salathé, 2015) dataset ordered (left–right) as in **Table 1**.

The *PlantVillage* dataset serves as a valuable resource for plant disease detection and research. Introduced in (Hughes and Salathé, 2015), this dataset emerged from a not-for-profit collaboration between Penn State University in the US and EPFL in Switzerland. In this project tens of thousands of plant leaf images were collected under controlled conditions. These leaf images were carefully annotated and made openly and freely accessible. The resulting dataset comprises 54 303 leaf images across 38 classes, including 14 crop species and 26 diseases, featuring both healthy and unhealthy leaves. **Figure 1** shows an example for each of the 38 classes in the dataset and **Table 1** shows the name of the classes together with the number of images per class. It is observed that the dataset is highly unbalanced.

**Table 1:** Classes and number of images per class in the *PlantVillage* dataset (Hughes and Salathé, 2015).

Crop	Disease	Images	Crop	Disease	Images
Apple	scab	630	Pepper	healthy	1478
Apple	black rot	621	Potato	early blight	1000
Apple	cedar rust	276	Potato	healthy	152
Apple	healthy	1645	Potato	late blight	1000
Blueberry	healthy	1502	Raspberry	healthy	371
Cherry	healthy	854	Soybean	healthy	5090
Cherry	powdery mildey	1052	Squash	powdery mildew	1835
Corn	cercospora	513	Strawberry	healthy	456
Corn	rust	1192	Strawberry	leaf scorch	1109
Corn	healthy	1162	Tomato	bacterial spot	2127
Corn	northern leaf blight	985	Tomato	early blight	1000
Grape	black rot	1180	Tomato	healthy	1592
Grape	black measles	1384	Tomato	late blight	1910
Grape	healthy	423	Tomato	leaf mold	952
Grape	isariopsis leaf spot	1076	Tomato	septoria leaf spot	1771
Orange	citrus greening	5507	Tomato	spider mites	1676
Peach	bacterial spot	2291	Tomato	target spot	1404
Peach	healthy	360	Tomato	mosaic virus	373
Pepper	bacterial spot	997	Tomato	yellow leaf curl	5357

Researchers and developers can leverage this dataset to improve crop health monitoring and disease diagnosis. Actually, this dataset has been widely used for research on plant disease detection using deep learning techniques (Mohameth et al., 2020; Argüeso et al., 2020).

Despite the good results obtained by training deep learning models with this dataset, it is a very challenging dataset for extracting features with backbones pre-trained on general data. The reason is that the dataset is very specific. All the images in the dataset show one green leaf over a similar background. Shapes, colors and textures are very similar through the complete dataset. The characteristics that differentiate the classes are quite subtle in many cases: the differences in the shape of the leaves may distinguish the crop and the colors, shapes and textures of the brownish or yellowish spots on the leaves may distinguish the disease.

### 2.2 *Oxford 102 flower* dataset

**Figure 2:**
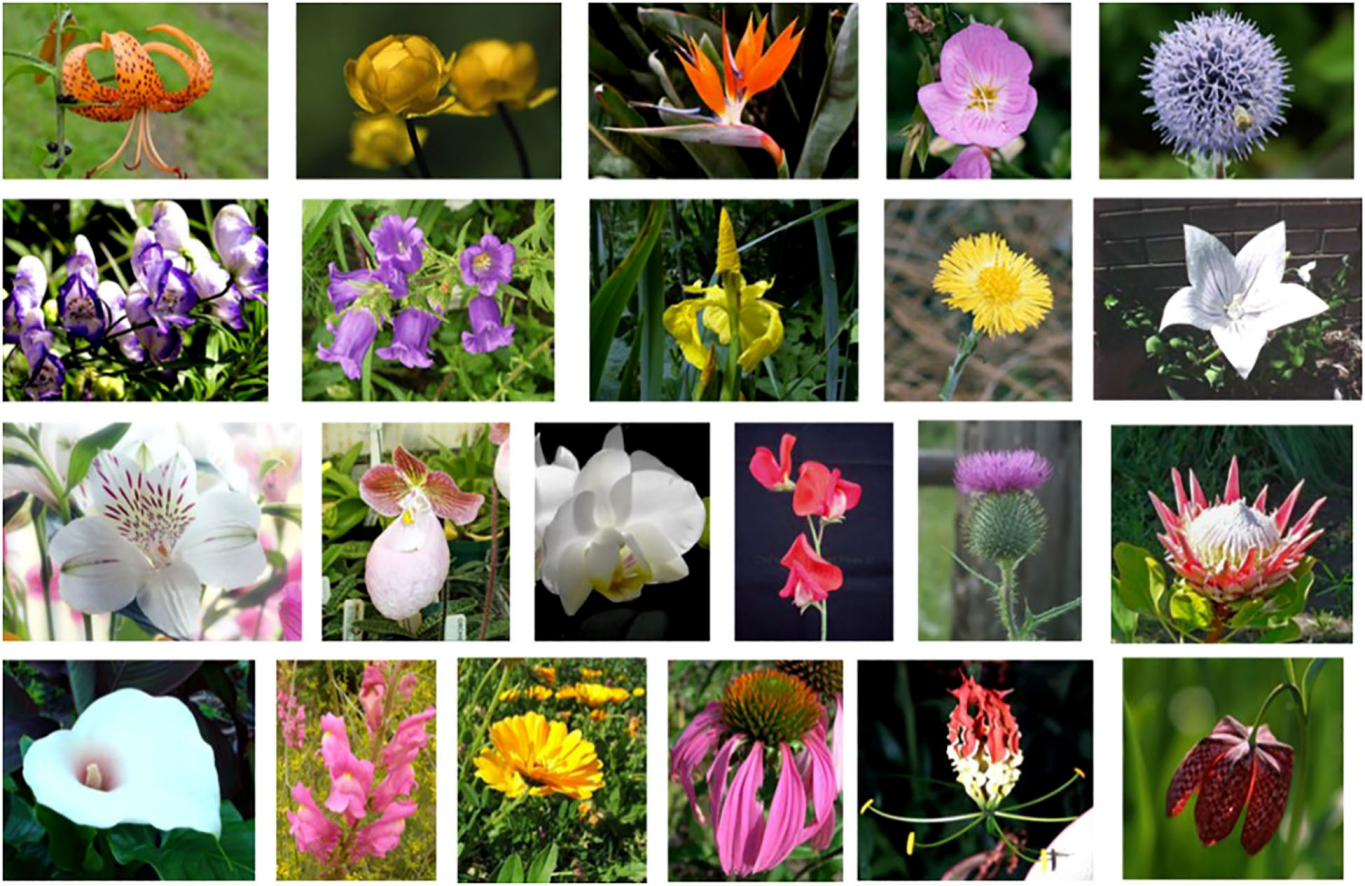
Examples from 22 of the 102 classes in the Flowers dataset (Nilsback and Zisserman, 2008).

The *Oxford 102 Flower* dataset is a valuable resource for image classification tasks introduced in (Nilsback and Zisserman, 2008). The dataset consists of 8 189 images of 102 flower categories, with each category representing a commonly occurring flower in the United Kingdom. Within each category, there are between 40 and 258 images. Most of these images were collected from the web, while a smaller number were acquired directly by the authors. **Figure 2** shows examples of 22 of the categories in the dataset. It is observed that the images exhibit variations in scale, pose, and lighting. **Table 2** shows the name of the 102 categories in the dataset and the number of images per category. Similar to the *PlantVillage* dataset, the *Oxford 102 Flower* dataset exhibits significant class imbalance.

**Table 2:** Classes and number of images per class in the Flowers dataset (Nilsback and Zisserman, 2008).

Category	Images	Category	Images	Category	Images
Alpine sea holly	43	Buttercup	71	Fire lily	40
Anthurium	105	Californian poppy	102	Foxglove	162
Artichoke	78	Camellia	91	Frangipani	166
Azalea	96	Canna lily	82	Fritillary	91
Ball moss	46	Canterbury bells	40	Garden phlox	45
Ballon flower	49	Cape flower	108	Gaura	67
Barbeton daisy	127	Carnation	52	Gazania	78	
Bearded iris	54	Cautleya spicata	50	Geranium	114	
Bee balm	66	Clematis	112	Giant white arum lily	56	
Bird of paradise	85	Colt’s foot	87	Globe thistle	45	
Bishop of llandaff	109	Columbine	86	Globe-flower	41	
Black-eyed susan	54	Common dandelion	92	Grape hyacinth	41	
Blackberry lily	48	Corn poppy	41	Great masterwort	56	
Blanket flower	49	Cyclamen	154	Hard-leaved pocket orchid	60	
Bolero deep blue	40	Daffodil	59	Hibiscus	131	
Bougainvillea	128	Desert-rose	63	Hippeastrum	76	
Bromelia	63	English marigold	65	Japanese anemone	55	
King protea	49	Peruvian lily	82	Stemless gentian	66	
Lenten rose	67	Petunia	258	Sunflower	61	
Lotus	137	Pincushion flower	59	Sweet pea	56	
Love in the mist	46	Pink primrose	40	Sweet William	85	
Magnolia	63	Pink-yellow dahlia	109	Sword lily	130	
Mallow	66	Poinsettia	93	Thorn apple	120	
Marigold	67	Primula	93	Tiger lily	45	
Mexican aster	40	Toad lily	41	Prince of Wales feathers	40	
Mexican petunia	82	Purple coneflower	85	Tree mallow	58	
Monkshood	46	Red ginger	42	Tree poppy	62	
Moon orchid	40	Rose	171	Trumpet creeper	58	
Morning glory	107	Ruby-lipped cattleya	75	Wallflower	196	
Orange dahlia	67	Siam tulip	41	Water lily	194	
Osteospermum	61	Silverbush	52	Watercress	184	
Oxeye daisy	49	Snapdragon	87	Wild pansy	85	
Passion flower	251	Spear thistle	48	Windflower	54	
Pelargonium	71	Spring crocus	42	Yellow iris	49	

Researchers and developers use this dataset for benchmarking machine learning models. It serves as a challenging dataset due to the variations in flower appearance and the large number of classes. In fact, there is a large similarity between classes and, as the flowers are non-rigid objects that can deform in many ways and the same class of flower can have different color, there is also a large variation within classes. The extraction of relevant features is therefore challenging. Flower classes are sometimes distinguished by color, sometimes by shape or sometimes by patterns on the petals, but often inside a class there are examples of different colors or shapes. The intra-class variability is high and inter-class variability is low, which makes the characterization and class separability problem a challenge.

## 3 Methods

**Figure 3:**
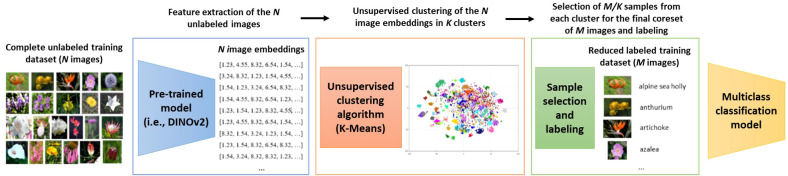
Flowchart of the complete proposed solution. There are three main steps: feature extraction, unsupervised clustering and selection of samples for the coreset. *N* is the number of images in the complete unlabeled training dataset, *K* the number of clusters calculated by K-Means and *M* the number of images in the final reduced labeled training dataset (coreset). The input of the pipeline are the *N* unlabeled images and the output the *M* images of the coreset that are labelled and used for the training of the classification model.

In this paper, we propose a simple but effective coreset selection method for agricultural use cases. The goal of coreset selection is to choose a small subset of images that accurately captures the diversity present in the entire dataset. Our proposed approach relies on a feature extractor, followed by an unsupervised clustering algorithm. From the resulting clusters, we select samples to form the final coreset and train the multiclass classification model. **Figure 3** shows a flowchart with the different steps of the complete proposed solution.

### 3.1 Feature extraction

An effective extraction of the relevant features of the images is key for the success of the proposed coreset selection method. We propose to extract the features using pre-trained backbones, which eliminates the need for *ad-hoc* feature extraction based on classical image processing or training deep learning models like autoencoders. Overall, this simplifies the coreset selection process significantly.

Until recently, pre-trained backbones were ill-suited for extracting relevant features from images in very specific application domains like agriculture. The available backbones were typically based on convolutional neural networks (CNNs) and trained using supervised methods on large public datasets such as ImageNet, which primarily contain generic object categories. However, recent advancements in image network architectures have introduced transformer-based neural networks (Dosovitskiy et al., 2020). These transformers can capture global information from images, surpassing the limitations of local feature extraction. Additionally, self-supervised learning has proven highly effective for training backbones. By avoiding manual annotation, self-supervised learning enables the use of significantly more data during training. It also eliminates annotator bias, allowing backbones to extract relevant features from entire images without category-specific constraints. The most successful self-supervised tasks are based on contrastive learning, which assumes that perturbations applied to unlabeled inputs should not alter the prediction (Chen et al., 2020; Zbontar et al., 2021; Caron et al., 2021). All this, together with the fact that nowadays there are sufficient computational resources to handle large amounts of data, has allowed to obtain foundation models that are trained in a self-supervised way using tons of data from a wide range of data sources. In our method, we propose to use one of these foundation models to extract the relevant features from the images. We demonstrate the importance of this choice by conducting experiments with various other pre-trained backbones.

Specifically, in our method we propose to use the recent DINOv2 (Oquab et al., 2023) as the pre-trained backbone for unsupervised feature extraction. DINOv2 significantly improves previous state-of-the-art image foundation models, making it an excellent choice for extracting the relevant features from images of a wide range of application fields. The vector produced by a pre-trained deep learning model, which represents the extracted features of an input image, is commonly referred to as the image embedding.

### 3.2 Unsupervised clustering algorithm

Unsupervised clustering methods aim to group similar data points together based on certain criteria. In our work, we propose to use an unsupervised clustering method to group the image embeddings extracted by the pre-trained deep learning models, such as DINOv2, into coherent regions. We propose to use K-Means as the unsupervised clustering algorithm, as it is a quite simple and widely used clustering algorithm. Given a set of data points (in our case image embeddings), K-Means split them into a specified number of clusters (indicated by ‘K’) such that the sum of squared distances between data points and their cluster centroids is minimized.

Key question for any clustering method is to have the relevant and meaningful features of the data to cluster. That is, the feature extraction is the most relevant part to get a good clustering. Therefore, for our method using a simple and widely used, but anyway effective, clustering algorithm as K-Means is enough to select a good coreset that represents the complete dataset.

One of the characteristics of K-Means is that it requires indicating beforehand the number of clusters (*K*) and this number is fixed. In our approach, we introduce various criteria for determining the optimal number of clusters. Specifically, we evaluate two main criteria: one based on the dataset’s class count (where the number of clusters equals the number of classes or a multiple thereof) and another based on the number of images in the final coreset aimed for manual annotation.

### 3.3 Selection of the samples for the coreset

After extracting the clusters, we select samples from these clusters to form the coreset, which will later be manually annotated. The desired coreset size is predetermined. Let *N* be the number of images in the complete unlabeled training dataset, *K* the number of clusters from K-Means, and *M* the number of images in the desired reduced labeled training set. At this stage of the coreset selection method workflow, we have *N* image embeddings clustered into *K* clusters, and we need to select *M* samples from these clusters.

When the number of clusters (*K*) equals the number of classes or a multiple thereof, we randomly select samples from each cluster until the desired coreset size (*M*) is achieved. This ensures that the coreset contains approximately the same number of samples from each cluster. Consequently, in this case, we randomly take *M/K* samples from each cluster for the coreset.

Alternatively, when the number of clusters (*K*) matches the desired number of images in the final coreset (*M*), that is, when *K=M*, we randomly choose one sample from each calculated cluster for the final coreset.

### 3.4 Multiclass classification model

The selected samples and their manual annotations are used for training and validating a multiclass classification model. We allocate 10% of the samples for validation, while the remaining 90% are used for training. The classification model architecture used in the experiments is always based on the well-known and extended ResNet (He et al., 2016) (either ResNet18 or ResNet50, depending on the specific experiment). The classification model is always trained for 300 epochs with a batch size of 16 and a learning rate of 0.001. The loss function is categorical crossentropy and the optimizer is Adam. The input size of the images is always 256x256 pixels and random horizontal and vertical flip augmentation is applied to the training samples before entering the model. Before training, the ResNet backbone is initialized with the weights obtained from pretraining with ImageNet. During training, the validation images are used to monitor the model’s performance on unseen data and to save the best weights, defined as those where the validation loss is minimized.

### 3.5 Experiments

To assess the effectiveness of the proposed coreset selection method, we compare the outcomes of reducing the training dataset using the proposed method with those obtained from random reduction. We conduct experiments on the two publicly available agricultural datasets described before: *PlantVillage* dataset and *Oxford 102 Flower* dataset. The target task of the models for both datasets is multiclass classification.

For the dataset formation in each experiment, we begin by setting aside 10% of the dataset samples for testing. These test samples are randomly selected from the complete dataset and are assumed to have the corresponding ground truth for calculating performance metrics. They are not part of the coreset selection workflow and are used solely for evaluation.

The remaining 90% of samples are then reduced either by randomly selecting a subset or by using the proposed coreset selection method with the corresponding configuration for the purpose of the experiment. In each round of experiments, we apply various percentages of reduction. **Table 3** displays the number of images in the training + validation set after excluding the testing samples and performing reductions for each dataset. The reductions applied to each dataset are determined based on the number of images and classes in that dataset.

**Table 3:** Percentages of the dataset size and the corresponding number of samples for each dataset of the training + validation set.

*PlantVillage*		*Oxford 102 Flower*	
Percentage of images	Number of images	Percentage of images	Number of images
100%	48875	100%	6633
30%	14662	30%	1990
10%	4887	20%	1327
5%	2443	15%	995
3%	1466	10%	663
1%	488	7%	464
0.5%	244	5%	332
0.3%	146	3%	199

To minimize the impact of randomness in the test set split, the image selection and the random initialization of the K-Means algorithm, we repeat all experiments three times. Specifically, we perform three repetitions of the test set separation and the application of dataset reductions across all tested configurations. For each experiment, we report the average metrics of the three repetitions along with the standard deviation.

The result of each experiment is evaluated by the metrics obtained for the test images when training the multiclass classification model with the reduced dataset in each case. The multiclass classification metric reported in each experiment is the macro-averaged F1 score. This score balances precision and recall through the harmonic mean between both metrics. It provides a comprehensive assessment of the classification model’s performance.

In our initial experiments, we aim to validate whether reducing the dataset using the proposed coreset selection method yields better results than random reduction. Additionally, we investigate the optimal strategy for the proposed method in terms of the number of clusters to extract using K-Means. For these experiments, we keep the DINOv2 model fixed as the backbone for feature extraction. In the second round of experiments, the goal is to assess the influence of the pre-trained backbone used for feature extraction in the performance of the proposed coreset selection method. The main objective of this analysis is to assess the significance of using a large foundational model like DINOv2 for the proposed method. For that, we fix the best strategy of number of clusters of the K-Means obtained from the previous experiments. We compare results by reducing the number of training samples randomly and by applying the proposed coreset selection method with various pre-trained backbones, all having similar number of parameters.

## 4 Results

In this section the results of the performed experiments are presented.

### 4.1 Random vs coreset selection with different number of clusters

To validate the proposed method, we select for labelling a reduced number of samples from the complete unlabeled training dataset using both random selection and the proposed coreset selection method. We then train the multiclass classification model with these reduced labeled training datasets and compare the F1 score obtained for the same test images with the trained models. Additionally, to determine the optimal number of clusters to extract using K-Means, we test different values for the number of clusters (*K*) in the proposed coreset selection method. Specifically, we conduct experiments with *K* equal to the number of images selected for the labeled reduced training set, *K* equal to the number of classes in the dataset, and *K* equal to double the number of classes in the dataset.

**Figure 4:**
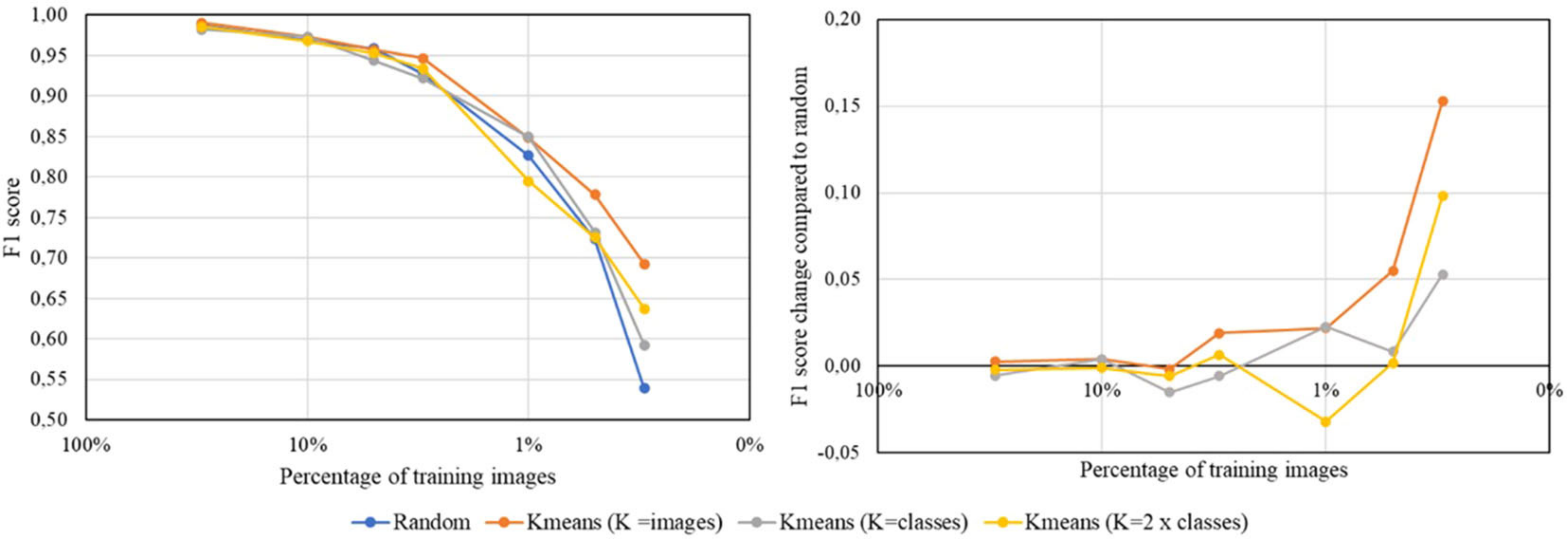
Results obtained training the multiclass classification model using a ResNet18 backbone with the PlantVillage dataset. The graphs use different colors to indicate random reduction and reduction by the proposed coreset selection method with varying number of clusters (K=number of images in the labeled reduced dataset, K=number of classes in the dataset and K=double the number of classes). The left plot displays the absolute F1 scores achieved for each size of the reduced dataset, while the right plot shows the difference in F1 scores compared to those obtained with random reduction, highlighting the improvements from the proposed coreset selection method.

**Figure 4** illustrates the results for the PlantVillage dataset, demonstrating that the classification model trained with the reduced dataset selected by the proposed coreset selection method generally outperforms the model trained with the randomly reduced dataset across various sizes of the selected training and validation datasets.

A detailed analysis of the *PlantVillage* dataset results is shown in **Table 4** and reveals that when the dataset reduction is minimal, the outcomes are similar whether the reduced training and validation subsets are selected randomly or using the proposed method. However, as the dataset size decreases, the impact of the proposed selection method becomes more significant. Specifically, when up to 5% of the data (2 443 images) is used for training and validation, there is no noticeable difference between random selection and the proposed method. For larger reductions, the proposed method’s contribution becomes increasingly evident, leading to improvements of up to 0.15in the F1 score. For example, when the dataset is reduced to 0.3% of its original size, the F1 score achieved with random reduction is 0.54, whereas the proposed coreset selection method boosts it to 0.69. Additionally, it is observed that for larger reductions, the proposed coreset selection method provides more consistent results than random reduction. This is evidenced by the smaller standard deviation between the results obtained in the three repetitions of the experiment when using the best configuration of our coreset selection method in bigger reductions.

**Table 4:** The mean and standard deviation of the F1 scores from classification models trained on various sizes of reduced training datasets using different strategies (random reduction and the proposed solution with varying numbers of clusters for K-Means) are reported.

Percentage of reduction in the training dataset		30%	10%	5%	3%	1%	0.5%	0.3%
**ResNet18**	Random	0.99 ± 0.00	0.97 ± 0.01	**0.96 ± 0.00**	0.93 ± 0.01	0.83 ± 0.01	0.72 ± 0.04	0.54 ± 0.05
	K=classes	0.98 ± 0.01	0.97 ± 0.00	0.94 ± 0.02	0.92 ± 0.01	0.85 ± 0.01	0.73 ± 0.06	0.59 ± 0.05
	K=2·classes	0.99 ± 0.01	0.97 ± 0.00	0.95 ± 0.02	0.93 ± 0.01	0.80 ± 0.01	0.73 ± 0.06	0.64 ± 0.05
	K=images	**0.99 ± 0.00**	**0.97 ± 0.00**	0.96 ± 0.01	**0.95 ± 0.01**	**0.85 ± 0.00**	**0.78 ± 0.02**	**0.69 ± 0.03**
**ResNet50**	Random	0.98 ± 0.01	**0.98 ± 0.00**	**0.96 ± 0.01**	0.94 ± 0.02	0.84 ± 0.02	0.74 ± 0.05	0.55 ± 0.03
	K=classes	**0.99 ± 0.01**	0.97 ± 0.01	0.96 ± 0.01	0.93 ± 0.01	0.84 ± 0.04	0.74 ± 0.05	0.64 ± 0.01
	K=2·classes	0.98 ± 0.01	0.97 ± 0.01	0.95 ± 0.02	0.94 ± 0.02	0.81 ± 0.03	0.72 ± 0.03	0.66 ± 0.02
	K=images	**0.99 ± 0.01**	0.98 ± 0.00	0.96 ± 0.02	**0.95 ± 0.02**	**0.86 ± 0.02**	**0.79 ± 0.03**	**0.67 ± 0.02**

The results include models based on both ResNet18 and ResNet50 backbones. Bold values remark the experiments showing the best results for every proposed reduction in the training data and the network architecture.

When determining the optimal number of clusters, two main strategies are compared: selecting the number of clusters based on the desired number of images in the coreset or based on the number of classes in the dataset. The results in the table show that, in general, the proposed solution outperforms random reduction regardless of the clustering strategy used. However, the findings indicate that the best approach is to set the number of clusters equal to the number of images in the coreset, and then select one image from each cluster to form the coreset for training the classification model. This strategy yields significant improvements, especially for the smallest training and validation sets. For example, training the classification model with only 0.3% of the available images (146 images) results in an F1 score of 0.54 when samples are selected randomly, compared to an F1 score of 0.69 when using the coreset selection method with 146 clusters in K-means. This behavior is logical, as the K-means algorithm aims to find cluster centroids that are as far apart as possible while keeping the samples within the same cluster as close as possible. We need our selected samples to be as diverse as possible, which is better achieved by having the number of clusters equal to the number of images we want to select.

To study the influence of classification model size on the results, in **Table 4** we also compare the outcomes of models based on ResNet18 and ResNet50. It is well-known that larger neural networks are more suitable for bigger datasets, as they have more information to learn from. However, for smaller datasets, smaller neural networks are preferable to avoid overfitting. In our experiments, the dataset size varies significantly (from 14,662 images for training and validation to just 146 images). Therefore, to confirm that network size does not influence the results, we conduct experiments with two different network sizes. The data in **Table 4** shows that the F1 scores obtained with both backbones are very similar in all cases, with differences not exceeding 0.01 in nearly all instances. This means that for both tested backbones the conclusions are the same: the proposed coreset selection method outperforms random selection for smaller coreset sizes, and the best strategy is to set the number of K-means clusters equal to the number of images in the coreset. Thus, it can be concluded that using a smaller network is more efficient, as training process is faster and the performance of the classification model is not compromised, even outperforming larger networks when the training dataset is smaller.

**Figure 5:**
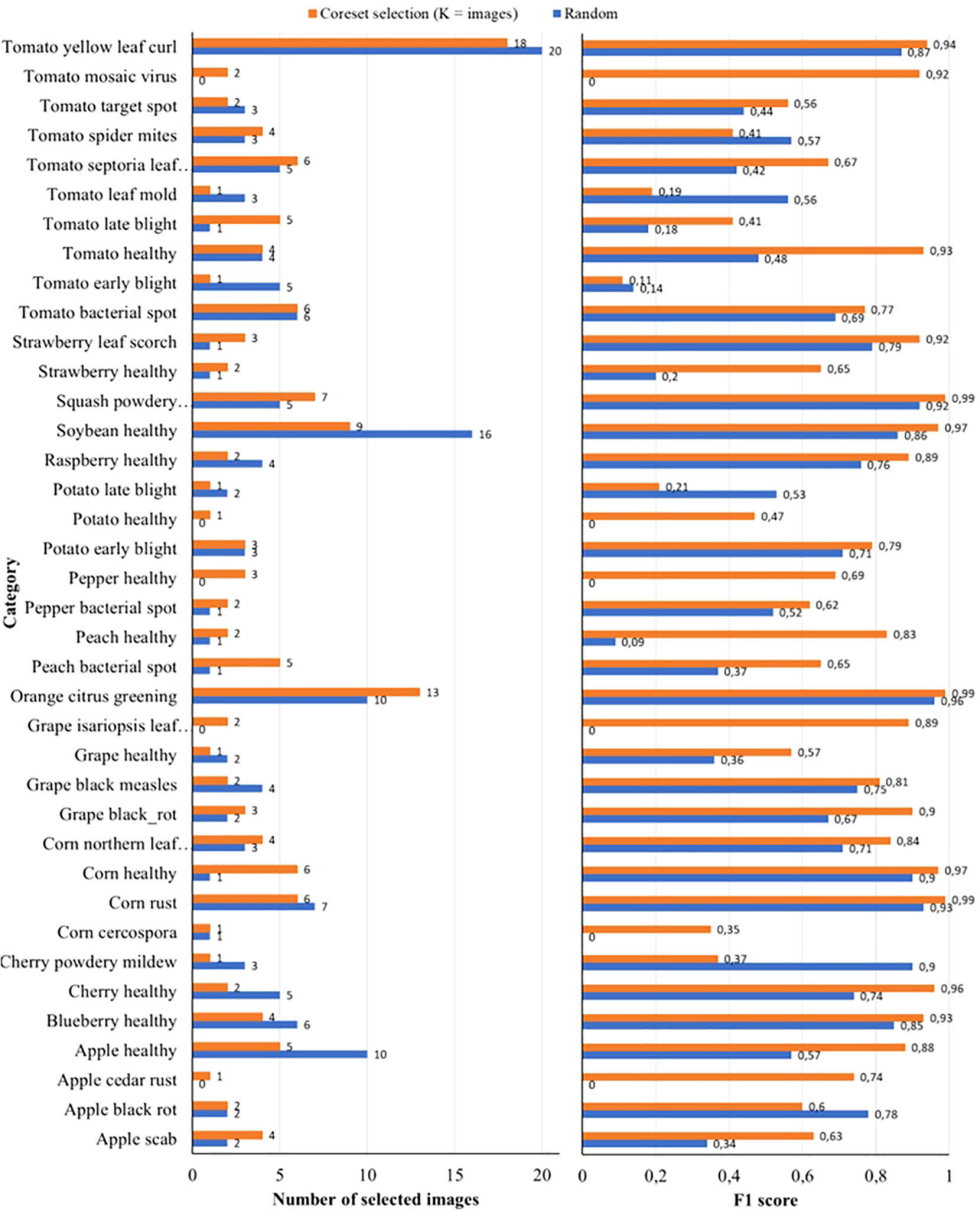
On the left number of images per class when reducing the training and validation sets of PlantVillage to 146 images by random reduction and by the proposed coreset selection method with the best strategy of number of clusters equal to number of classes. On the right the F1 score per class in each case.

**Figure 6:**
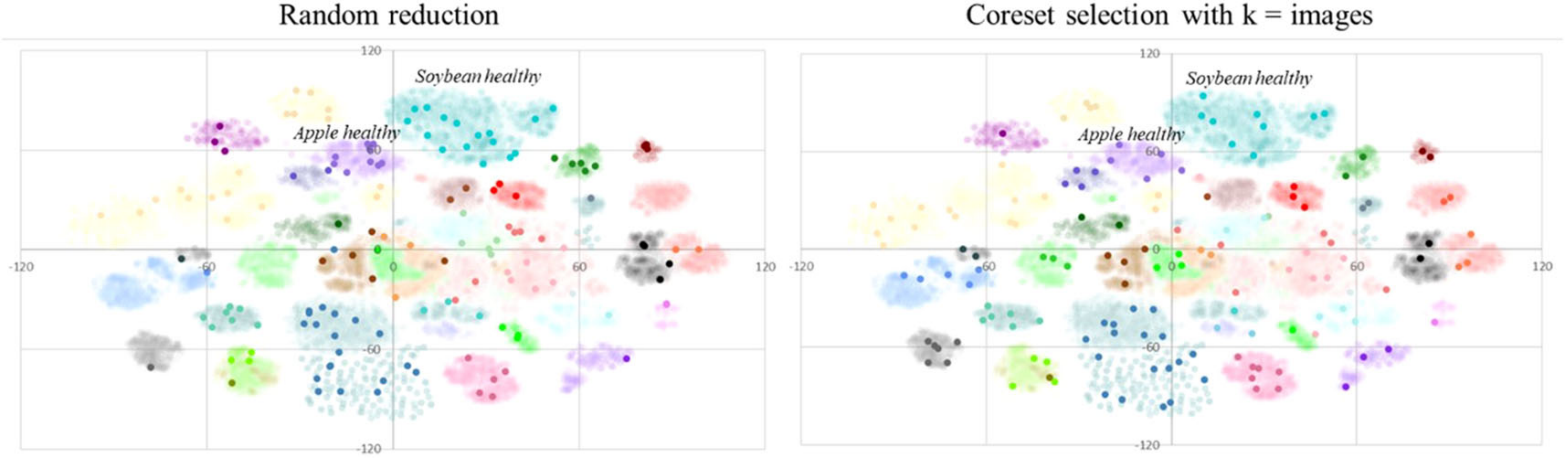
t-SNE representation of the complete PlantVillage dataset with the 146 images selected for training and validation on one of the experiments highlighted. On the left, the selection of the samples is done randomly and on the right the selection is done by the proposed coreset selection method with the best configuration.

To better understand why the proposed coreset selection method outperforms random reduction, we conducted an analysis in a scenario with significant reduction. Specifically, we examined the dataset balancing and distribution of the reduced datasets, each containing 146 images, generated by the proposed coreset selection method (with *K*=146) and by the random reduction method. **Figure 5** illustrates the number of images per class selected by both methods together with the F1 score per class in both cases and **Figure 6** shows the distribution of the selected samples in both cases in a t-SNE representation (der Maaten and Hinton, 2008) of the complete dataset. It is a two-dimensional spatial representation of the embeddings obtained from DINOv2 for all the samples of the dataset, colored by the real class to what each sample belongs and with the samples selected after the reduction highlighted.

It is clear that the proposed method achieves better dataset balancing compared to random reduction. This leads to an improved F1 score for nearly all classes. Notably, with random reduction, five classes have no selected images, resulting in an F1 score of 0 for these classes. In contrast, the proposed method ensures at least one image is selected for every class, significantly enhancing the F1 score for those classes. For instance, in the random reduction, no images are selected for classes such as “*Tomato mosaic virus*” or “*Grape isariopsis leaf* sp*ot*”. However, with the proposed method, two images per class are selected for these classes, improving their F1 scores from 0 to 0.92 and 0.89, respectively. The improved performance in dataset balancing achieved by the coreset selection method is due to the combination of image embeddings extracted by DINOv2 and the K-Means algorithm. This combination effectively separates images of different classes into distinct clusters based on their representative features. This is particularly important for unbalanced datasets like the *PlantVillage* dataset, where some classes are underrepresented.

Additionally, it is observed that the samples selected by the proposed method are better distributed across the entire dataset compared to those chosen by random reduction. This means that the samples selected in the proposed method represent better the variability of the whole dataset, enabling the model to learn to work in a wider scope of the use case. The improved variability is evident even within individual classes. For instance, in the “*Soybean healthy*” and “*Apple healthy*” classes (turquoise and purple samples in **Figure 6**, respectively), although the proposed method selected half as many samples as the random reduction, the F1 scores significantly improved. Specifically, the F1 score for “*Soybean healthy*” increased from 0.86 to 0.97, and for “*Apple healthy*,” it rose from 0.57 to 0.88. The reason is that, as it is observed in the t-SNE representation, the selected samples are better distributed across all the variability of the class.

**Figure 7:**
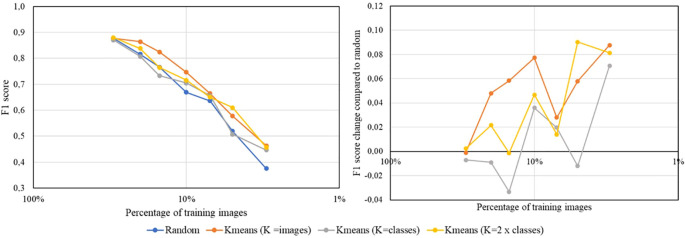
Results obtained training the multiclass classification model using a ResNet18 backbone with the Flowers dataset. The graphs use different colors to indicate random reduction and reduction by the proposed coreset selection method with varying number of clusters. The left plot display the absolute F1 scores achieved for each size of the reduced dataset, while the right plots show the difference in F1 scores compared to those obtained with random reduction, highlighting the improvements from the proposed coreset selection method.

Finally, to thoroughly evaluate the effectiveness of the proposed coreset selection method, **Figure 7** and **Table 5** presents the results from the Flowers dataset. The conclusions drawn from these results are consistent with those from the PlantVillage dataset. Specifically, in the case of this dataset the proposed coreset selection method successfully selects representative samples from the entire dataset, yielding better results than random selection. Additionally, as in the case of PlantVillage the strategy of setting the number of clusters equal to the number of images to be selected generally proves to be the most effective.

**Table 5:** The mean and standard deviation of the F1 scores from classification models trained on various sizes of reduced training datasets using different strategies (random reduction and the proposed solution with varying numbers of clusters for K-Means) are reported.

Percentage of reduction in the training dataset		30%	20%	15%	10%	7%	5%	3%
**ResNet18**	Random	0.88 ± 0.01	0.82 ± 0.03	0.77 ± 0.04	0.67 ± 0.02	0.64 ± 0.03	0.52 ± 0.04	0.38 ± 0.03
	K=classes	0.87 ± 0.04	0.81 ± 0.05	0.73 ± 0.12	0.71 ± 0.06	0.66 ± 0.03	0.51 ± 0.09	0.45 ± 0.03
	K=2·classes	**0.88 ± 0.01**	0.84 ± 0.02	0.77 ± 0.03	0.72 ± 0.02	0.65 ± 0.02	**0.61 ± 0.05**	0.46 ± 0.02
	K=images	0.88 ± 0.02	**0.86 ± 0.02**	**0.83 ± 0.02**	**0.75 ± 0.02**	**0.66 ± 0.02**	0.58 ± 0.04	**0.46 ± 0.03**

Bold values remark the experiments showing the best results for every proposed reduction in the training data and the network architecture.

For the Flowers dataset, the benefits of the proposed method are evident even with smaller dataset reductions, as this dataset has fewer samples and more classes than PlantVillage. Using 10% of the dataset for training and validating the multiclass classification model involves only 663 images, which would equate to approximately 6 images per class in a perfectly balanced dataset. In this scenario, the F1 score improves from 0.67, when using 663 randomly selected images to train a classification model based on ResNet18, to 0.75 when the same number of images are selected using the proposed coreset selection method. These results confirm the generalizability of the proposed method across different use cases.

**Figure 8:**
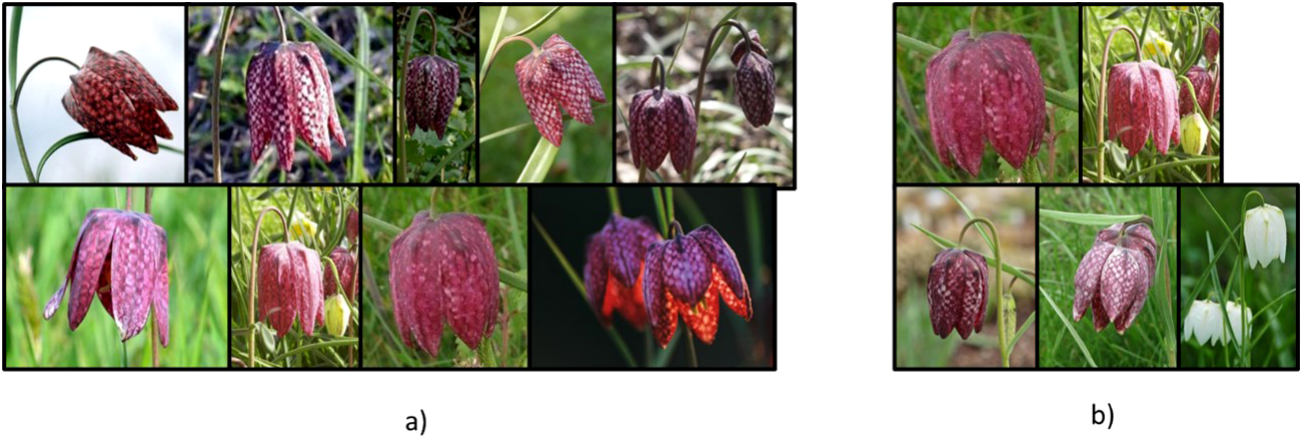
Images of “fritillary” class selected for training in one of the experiments with 10% of the dataset by **(a)** the random reduction and **(b)** the proposed coreset selection method with k equal to number of images.

In **Figure 8**, we analyze the images of one of the dataset’s classes selected by both the random reduction method and the proposed coreset selection method (with K equal to the number of images) in one of the experiments for training the classification model with 10% of the dataset. Specifically, we examine the selection for the “fritillary” class. We observe that the random reduction method selects 9 images of this class, while the proposed method selects 5 images. Notably, this type of flower can be either pink or white in the dataset. Despite selecting more images, the random reduction method fails to include any white flowers. In contrast, the coreset selection method ensures that at least one white flower is included. This results in an improvement of the F1 score for this class from 0.82 to 1 with half the training samples. This demonstrates how the proposed coreset selection method better represents the dataset’s variability in the reduced dataset.

### 4.2 Comparison of pre-trained backbones for feature extraction

**Figure 9:**
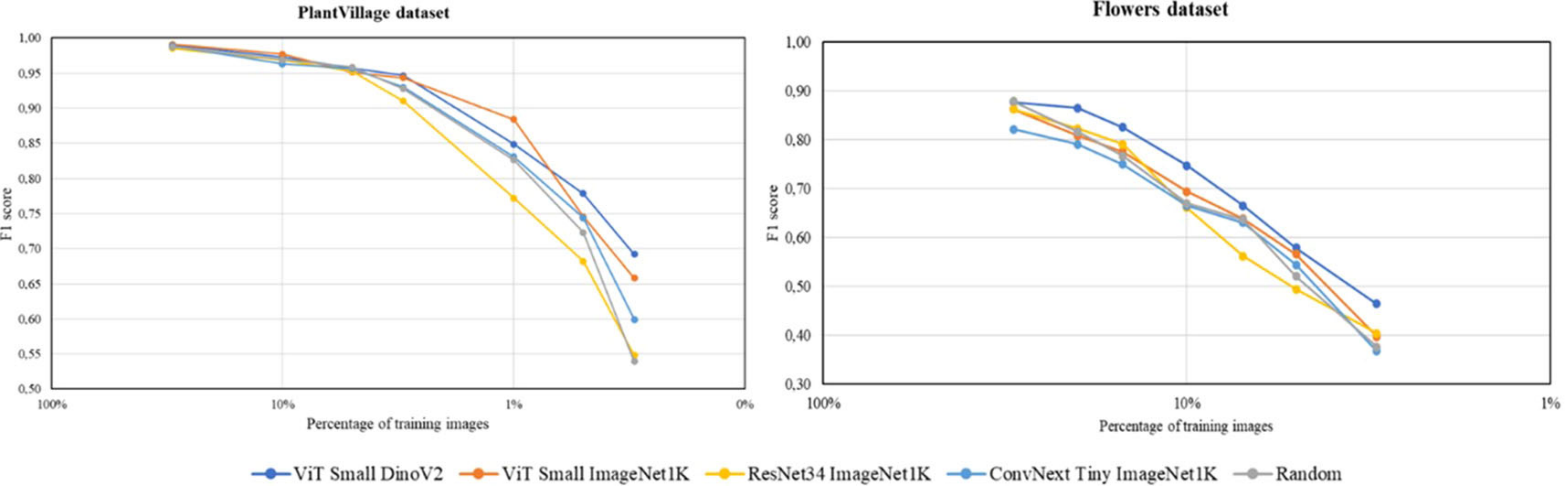
Results for the PlantVillage dataset (left) and Flowers dataset (right) comparing various pre-trained backbones used as feature extractors. The plots illustrate the F1 score of the multiclass classification model, trained with different percentages of the training and validation datasets. Each color represents the results obtained using the proposed coreset selection method for dataset reduction with different feature extractors, except for the grey color, which shows the results obtained by randomly reducing the dataset.

To evaluate the significance of the selected pre-trained backbone for extracting image features in the proposed coreset selection method, the method is tested using various pre-trained backbones with a similar number of parameters. Specifically, we consider ResNet34 (He et al., 2016) (a traditional convolutional architecture) and the ConvNext-tiny (Liu et al., 2022) (a more modern convolutional architecture), both pre-trained in a supervised way using the 1.28M images of ImageNet-1K. Additionally, we include ViT-Small (Dosovitskiy et al., 2020), which is transformer-based, also pre-trained on ImageNet-1K in a supervised manner. Finally, we consider DINOv2 (Oquab et al., 2023), based also on a ViT-Small architecture but pre-trained in a self-supervised way using 142M images. Throughout these experiments, ResNet18 is used as the backbone for the multiclass classification model and the best configuration from previous experiments is fixed, that is, the number of clusters in K-means is set equal to the number of images to select. The classification performance with different sizes of training and validation sets is reported for both datasets. **Figure 9** shows the results obtained in this comparison.

The results from both datasets lead to similar conclusions. On the one hand, the worst outcomes are observed when using the proposed coreset selection method extracting the image features with convolutional architectures, such as ResNet34 or ConvNext Tiny, pre-trained in a supervised manner on ImageNet-1K. Specifically, when using ResNet34, a more traditional backbone, the results generally do not even surpass those obtained by randomly reducing the dataset. However, switching to more modern architectures, such as the transformer-based network ViT Small, yields better results. These findings suggest that transformer-based networks are more effective at extracting image features than convolutional networks. When both types of architectures, of similar size, are pre-trained with the same data and approach (supervised), the transformer-based architecture consistently provides superior results.

On the other hand, it is observed that using the same transformer-based architecture for image feature extraction yields better results when it has been pre-trained in a self-supervised manner rather than a supervised way. For the Flowers dataset, the self-supervised approach consistently produces much better results. In the case of the PlantVillage dataset, better performance is mainly observed when the dataset reduction is more significant. The superior results obtained with the self-supervised pre-trained backbone can be attributed to the possibility to use much more data in the pre-training process, as manual annotation is not required. In fact, in the supervised pre-training 1.28M images are used while DINOv2 is pre-trained in a self-supervised way using 142M images. This extensive pre-training allows the network to learn to extract features from a much larger number of examples, covering a wider spectrum of the reality and representing many more fields of application. Additionally, supervised pre-training introduces annotator bias, leading the model to primarily learn to distinguish objects. In contrast, self-supervised pre-training proposed in DINOv2 enables the model to learn from the entire image, focusing on important details and thus becoming a better image feature extractor.

To further understand the results obtained in these experiments, we calculate two clustering metrics using the real labels of the complete dataset and the different pre-trained backbones used for feature extraction. Specifically, we calculate the Davies-Bouldin score and the Silhouette score. The Davies-Bouldin score measures the average similarity ratio of each cluster with its most similar cluster, considering both the compactness within clusters and the separation between clusters. The best value for this score is 0. The Silhouette score measures how similar an object is to its own cluster compared to other clusters, with values ranging from -1 to 1, where 1 is the best value. In our case, better values of these scores indicate better separation of the different classes in the dataset, and consequently, better performance of the feature extraction. **Table 6** shows the results obtained for these metrics. They are consistent with those shown in **Figure 9**, with ViT Small DINOv2 being the pre-trained backbone that best clusters the samples of the same class in both datasets. This indicates that it is the backbone that most effectively extracts the relevant features of the images.

**Table 6:** Davies-Bouldin and Silhouette score for both complete datasets and the tested pre-trained backbones considering the real labels.

Percentage of reduction in the training dataset	PlantVillage dataset		Flowers dataset	
	Davies-Bouldin	Silhouette	Davies-Bouldin	Silhouette
ResNet34 ImageNet1K	27.43	-0.03	16.04	-0.05
ConvNext Tiny ImageNet1K	3.68	0.01	2.91	0.01
ViT Small ImageNet1K	3.21	0.04	3.13	0.04
ViT Small DINOv2	**2.90**	**0.09**	**1.40**	**0.30**

Bold values remark the experiments showing the best results for every proposed reduction in the training data and the network architecture.

**Figure 10:**
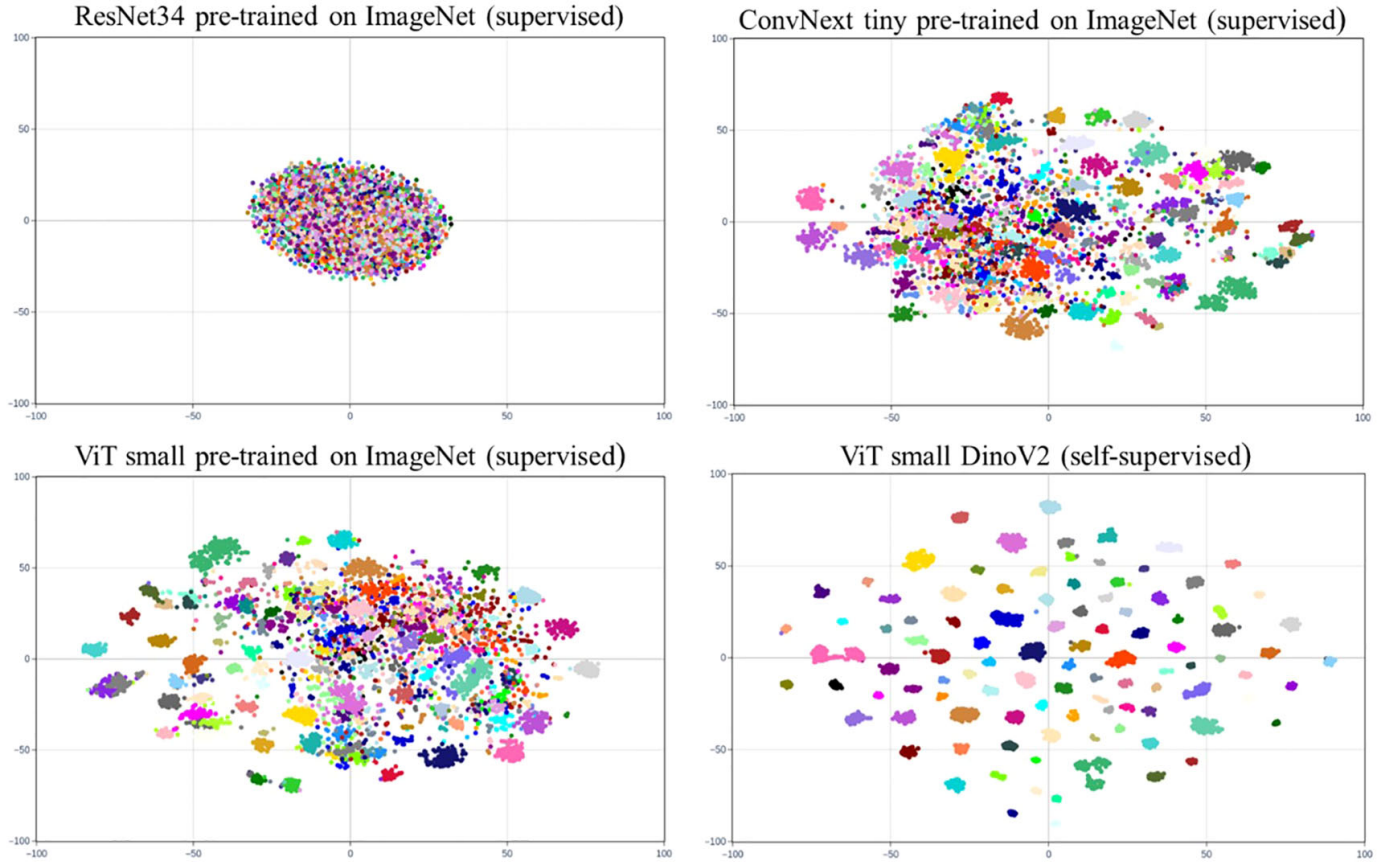
t-SNE representations of the feature vectors of the training and validation images of the Flowers dataset obtained by the selected pre-trained backbones. The samples are colored by the ground-truth class.

Additionally, to visually contrast these results we create t-SNE representations (der Maaten and Hinton, 2008) of the feature vectors produced by the tested pre-trained backbones for the Flowers dataset. This involves generating a two-dimensional spatial representation of the embeddings obtained by each pre-trained backbone for all the images available for training and validation, with each sample colored according to its ground truth class. **Figure 10** displays these representations. In the case of ResNet34 pre-trained in a supervised manner, the feature vectors are highly mixed, making it impossible to obtain proper clusters using K-Means. When switching to a more modern convolutional architecture like ConvNext Tiny, the feature vectors are better grouped by class, although they remain somewhat mixed overall. The results are very similar with a transformer-based architecture (ViT Small) pre-trained in a supervised way with the same data. Finally, DINOv2, which is also based on ViT Small but pre-trained in a self-supervised manner using a large amount of data, significantly enhances image feature extraction. The calculated embeddings are clearly grouped by class, allowing the K-Means algorithm to form clusters that accurately represent the dataset.

## 5 Conclusions

The coreset selection method proposed in this work is highly effective yet very simple to apply for choosing the most representative samples from a dataset for manual annotation. It enables the creation of a deep learning model for any agricultural use case with strong performance while significantly reducing the required annotation effort. The results obtained using this method to select the training set are significantly better than those achieved by random selection, especially when the reduction of annotated training data is substantial. This is particularly relevant for agricultural use cases where obtaining annotated images is usually challenging.

Additionally, this work also demonstrates the effectiveness of recent foundation models like DINOv2. Trained in a self-supervised manner on a vast amount of data, these models can properly extract the most relevant features from images. Proper feature extraction is critical for the effectiveness of the proposed coreset selection method.

Among the potential extensions and applications of this work, the proposed coreset selection method could be highly effective for selecting an initial set of samples to train a robust first model, which can then be improved through a continual active learning cycle. Having a high-performing initial model is crucial for the active learning cycle to yield better results. Additionally, this method could be suitable for selecting the few annotated data needed in a semi-supervised solution. Also, improvements in the method could be made, particularly in the unsupervised clustering part. While K-Means is a simple and effective algorithm, it has limitations, such as the need to specify the number of clusters in advance and the fact that it may not be the most suitable algorithm for a large number of clusters. Finally, it should be pointed out that, although the proposed method has been validated for multiclass classification on two different agricultural datasets, the concept could also be applied to select reduced training sets for any other task or use case, especially since the best configuration is to select K equal to the number of images and therefore the model task to apply this method is not limited to classification.

## Data Availability

Publicly available datasets were analyzed in this study. This data can be found here: https://www.robots.ox.ac.uk/~vgg/data/flowers/102/ and https://www.kaggle.com/datasets/mohitsingh1804/plantvillage.
